# Feasibility of Using a Community-Supported Agriculture Program to Improve Fruit and Vegetable Inventories and Consumption in an Underresourced Urban Community

**DOI:** 10.5888/pcd10.130053

**Published:** 2013-08-15

**Authors:** Sara A. Quandt, Janae Dupuis, Caitlin Fish, Ralph B. D’Agostino

**Affiliations:** Author Affiliations: Janae Dupuis, Caitlin Fish, and Ralph B. D’Agostino, Jr, Wake Forest School of Medicine, Winston-Salem, North Carolina. Dr Quandt and Dr D’Agostino, Jr, are also affiliated with the Wake Forest University Comprehensive Cancer Center, Winston-Salem, North Carolina. Dr Quandt is also affiliated with the Wake Forest University Translational Science Institute and the Maya Angelou Center for Health Equity at the Wake Forest School of Medicine.

## Abstract

**Introduction:**

Direct-to-consumer marketing efforts, such as community-supported agriculture (CSA), have been proposed as a solution for disparities in fruit and vegetable consumption. Evaluations of such efforts have been limited. The objective of this study was to test the feasibility of a CSA intervention to increase household inventory of fruits and vegetables and fruit and vegetable consumption of residents of an underresourced community.

**Methods:**

For this randomized, controlled feasibility study, we recruited 50 low-income women with children. Intervention (n = 25) participants were offered 5 educational sessions and a box of fresh produce for 16 weeks; control participants were not offered the sessions nor were they included in the produce delivery. We collected data on participants’ home inventory of fruits and vegetables and on their consumption of fruits and vegetables at baseline (May 2012) and postintervention (August and September 2012).

**Results:**

Of 55 potential participants, 50 were enrolled and 44 were reached for follow-up. We observed a significant increase in the number of foods in the household inventory of fruits and vegetables in the intervention group compared with the control group. The intervention group reported greater increases in fruit and vegetable consumption; however, these did not reach significance. Intervention participants picked up produce 9.2 (standard deviation = 4.58) of 16 weeks; challenges included transportation and work schedules. Most participants (20 of 21) expressed interest in continued participation; all stated a willingness to pay $10 per week, and some were willing to pay as much as $25 per week.

**Conclusion:**

CSA is a feasible approach for providing fresh fruits and vegetables to an underresourced community. Future studies should evaluate the impact of such a program in a larger sample and should take additional steps to facilitate participation.

## Introduction

Fruit and vegetable consumption helps prevent chronic diseases responsible for the major causes of illness and death in the United States (1–3). Despite the known benefits of eating fruits and vegetables, the typical US diet fails to meet recommendations (1). Substantial disparities in fruit and vegetable consumption exist by region, race, and income (4); these are mirrored in disparities in health outcomes and disease prevalence (5,6).

Limitations in community food sources and lack of transportation to reach food outlets may be responsible for some disparities in fruit and vegetable consumption (7–10). Residents of minority communities experience more problems in accessing healthful foods than do residents of predominantly white neighborhoods (11–14).

Suggested solutions to disparities in food access include direct-to-consumer marketing efforts, such as farmers markets, mobile produce trucks, community gardens, and community-supported agriculture (CSA) shares (15). CSA allows community residents to purchase local food directly from a farmer by buying a “share” of the farm’s anticipated production at the beginning of the growing season. Shareholders receive a box of produce each week. The required lump sum payment often restricts CSA programs to high-income consumers. Although some variations on CSA programs developed for low-income families have been described (16,17), their evaluation has been limited (18).

The primary objective of this pilot feasibility study was to test whether a program providing a summer’s CSA share and supportive programming around food purchase and food preparation would be associated with increased fruit and vegetable household inventory variety and increased fruit and vegetable consumption in low-income, minority families. Data are being used to determine programming revisions necessary to design a larger trial.

## Methods

### Study design and recruitment

We used a modified randomized design, conducted from May 2012 through September 2012, to test the feasibility of our intervention. All procedures were reviewed and approved by the Wake Forest School of Medicine institutional review board (IRB). The program, Farm Fresh Healthy Living, was developed, administered, and evaluated by a partnership of university researchers, a community nonprofit agency, and a noncertified organic farm.

Participants were clients of a nonprofit [501(c)3] community action agency in Forsyth County, North Carolina, who lived throughout the 413 square miles of the county. Over two-fifths (41.4%) of the county population is nonwhite or Hispanic (19). The county forms part of the Winston-Salem Metropolitan Statistical Area and has a population density of 859.2 persons per square mile. The agency conducts programs focused on family financial self-sufficiency and housing to help families with household incomes below 200% of the federal poverty guidelines to achieve self-sufficiency. Eligibility criteria were being 1) female, 2) at least 18 years old, 3) head of the household with at least 1 minor child, and 4) able to speak and understand English.

Typical randomization procedures were modified after extensive discussions with the agency leadership and personnel, who were reluctant to recruit women for a study in which they could possibly receive free food and then be randomized to a control condition. To alleviate this concern, a procedure to preserve the random assignment but allow recruitment for a specific condition (intervention or control) was devised and approved by the IRB. Agency personnel first produced a list of all eligible clients (N = 93), which was randomized by the university researchers to 2 lists. A random procedure was used to classify 1 list as intervention and the other as control. Names on each list were then placed in random order. Agency personnel trained by the researchers and using scripts approved by the IRB called participants in the order listed until 25 on each list had been recruited. Both intervention and control scripts recruited women to participate in research “designed to learn more about foods families in [the agency’s] programs eat, where they get these foods, and some of the factors involved in their choosing these foods.” Both scripts described the evaluation questionnaires. The intervention script described participation in the CSA program; the control script did not. Recruiters kept records of the disposition of each person on the list. From 29 potential intervention participants contacted, 1 was ineligible and 2 refused to participate (refusal ratio = 7%). From the list of 26 potential control participants contacted, 1 person was ineligible and none refused to participate. All who agreed to participate signed an authorization to have their contact information given to the researchers.

### Study intervention

The Farm Fresh Healthy Living Program provided intervention families with a CSA share from the farm, consisting of a three-quarter bushel box containing a 12- to 15-pound share of seasonal fruit and vegetable items, once each week for 16 weeks from May through August 2012. Boxes were available for pick-up every Thursday between 12:00 pm and 6:00 pm from the agency offices. Late pick-ups were accommodated with advance notice when possible. The box contained at least 2 simple recipes focused on the week’s produce, with emphasis on items that may have been unfamiliar to participants. The CSA shares were discounted by the farm and purchased by the community agency using private donations. During the program, the intervention group was offered 5 evening education and skill-building sessions: 3 cooking classes conducted by local North Carolina Cooperative Extension staff and based on the *Cook Smart, Eat Smart* curriculum (20); a tour of the participating farm; and a grocery store tour with a dietitian focused on healthful eating on a budget.

### Study evaluation and data analysis

Participants in intervention and control groups were interviewed twice by telephone: at baseline before the study began and again at follow-up after program completion. Interviews lasted 25 to 30 minutes and were conducted by an interviewer not connected with the project. This interviewer obtained verbal informed consent before collecting any study data. Participants received a $10 gift card for each interview completed. Both interviews obtained data for outcome analysis. The baseline interview included questions on personal characteristics; the follow-up interview included questions for process evaluation.

The independent variable was assignment to intervention or control group. Covariates collected included the following demographic characteristics: age, marital status, number of people in the household, years of education, race/ethnicity, and employment status. Self-efficacy for fruit and vegetable consumption was measured using a 5-item scale, ranging from “not at all confident” to “very confident” (21).

Two primary outcomes were measured. Home availability of fruits and vegetables (F&V availability) was measured using a checklist including fruit (14 items) and vegetables (25 items) (22). Items in any form — fresh, frozen, canned, or dried — were counted. Total F&V availability was obtained by summing all items; possible values ranged from 0 to 39. Fruit and vegetable intake (F&V intake) of the study participant was measured using the Centers for Disease Control and Prevention’s Behavioral Risk Factor Surveillance System (BRFSS) (23). Four questions queried the number of times in the past week a respondent had consumed fruit, dark-green vegetables, orange-colored vegetables, or other vegetables. The measure of total F&V intake was constructed by summing times per week for all 4 questions.

Process data from study records included the number of times produce was picked up during the program and the number of classes and events attended (range, 0–5). Process data from intervention participants included their reports of barriers to program participation, disposition of the food received, future willingness to provide partial payment for the program, and overall program assessment. Other process data came from debriefing meetings with agency, farm, and program staff.

Demographic characteristics of the intervention and control groups were compared by using counts and percentages. Differences were assessed by using χ^2^, Fisher exact, and *t* tests, as appropriate. General linear models predicting follow-up outcomes were constructed, adjusting for age, education, baseline values of the outcome of interest, and self-efficacy for fruit and vegetable consumption. *P* values equal to or less than .05 were considered significant, and all calculations were performed using SAS Version 9.2 (SAS Institute, Inc, Cary, NC).

## Results

Twenty-five women were recruited for each group (Table 1). At follow-up, we were able to contact and interview 23 control and 21 intervention group members. Participants ranged in age from 24 to 60; most were African American and unmarried. Educational attainment ranged from high school graduate (or general educational diploma) to graduate degree. No significant differences were observed between groups at baseline.

**Table 1:** Baseline Demographic Characteristics Of Participants in the Farm Fresh Health Living Feasibility Study, May 2012

Variable^a^	Total (n = 50)	Control (n = 25)	Intervention (n = 25)	*P* Value
**Mean age, y, (SD)**	37.34 (8.20)	38.16 (9.03)	36.52 (7.37)	.49
**Race**				
African American	48	24	24	.76
White or other	2	1	1	
**Marital status**				
Married	3	2	1	.50
Not married	47	23	24	
**Education, y**				
12–14	41	23	18	.07
15–17	9	2	7	

Abbreviation: SD, standard deviation.

a Values are presented as whole numbers unless otherwise indicated.

Intervention group participants picked up the produce box an average of 9.2 (standard deviation [SD] = 4.58; range, 1–16) of 16 weeks. They attended an average of 1.2 (SD = 1.32; range, 0–4) of the 5 events. We observed a significant increase in the number of foods in the total inventory of fruits and vegetables and in vegetables alone in the intervention group, compared with the control group (Table 2). We observed no significant increases in fruit and vegetable consumption in the intervention group, compared with the control group (Table 3), although results trended in favor of the intervention group having higher intakes.

**Table 2:** Self-Reports of Disposition of Foods in Participant Households

Food	Received^a^	Consumed^b^			Gave Away	Discarded
		Self	Child	Other		
Tomatoes	20	19	15	6	1	0
Carrots	16	14	12	7	1	0
Lettuce	19	19	14	5	0	0
Potatoes	20	20	19	8	0	0
Broccoli	18	17	13	8	0	0
Cucumbers	20	20	15	5	0	0
Onions	20	19	17	7	1	0
Cabbage	19	18	17	8	0	0
Zucchini/yellow summer squash	20	16	13	5	4	0
Herbs	18	15	13	5	2	1
Greens (such as kale, Swiss chard)	15	13	13	5	0	1
Beets	21	9	6	7	9	0
Green/wax beans	18	15	13	8	2	0
Corn	19	19	18	11	0	0
Garlic	15	15	12	5	0	0
Okra	17	11	10	5	5	0
Eggplant	11	7	4	1	4	0
Peppers	19	19	17	9	0	0
Cantaloupe/watermelon	19	18	18	11	0	0
Cherries	19	16	13	7	1	0
Berries, any type	13	12	10	4	0	0
Peaches	13	12	9	5	0	1

a *P* Foods available varied throughout the season. Therefore, the food each participant received depended on which weeks the participant picked up her box of produce.

b Participants who reported receiving a food were asked whether it was consumed in the household and by whom (self, child, other person), given away, or discarded.

**Table 3:** Participant Fruit and Vegetable Consumption and Household Inventory at Baseline (May 2010) and Follow-Up (August 2010)^a^

Variable	Control		Intervention		*P* Value^b^
	Baseline	Follow-up	Baseline	Follow-up	
**Fruit and vegetable inventory,^c^ n (SD)**					
Total	19.1 (5.4)	21.2 (5.9)	19.3 (5.8)	24.9 (4.8)	.02
Fruit	6.5 (2.9)	8.3 (2.0)	6.5 (2.7)	9.0 (2.0)	.32
Vegetables	12.6 (3.6)	12.9 (4.3)	12.9 (4.0)	15.9 (3.0)	.009
**Fruit and vegetable consumption, servings per week (SD)**					
Total	16.7 (12.9)	16.4 (8.1)	16.0 (9.2)	18.7 (6.2)	.17
Fruit	6.6 (7.8)	6.6 (4.9)	5.0 (3.8)	8.0 (4.5)	.40
Green vegetable	3.8 (2.8)	3.2 (2.0)	4.5 (3.3)	3.3 (2.4)	.90
Orange vegetable	1.2 (1.5)	1.1 (1.1)	2.3 (2.4)	1.8 (1.5)	.30
Other vegetable	5.1 (3.6)	5.5 (4.4)	4.2 (3.7)	5.6 (3.2)	.62

Abbreviation: SD, standard deviation.

a Analysis based on 23 control and 21 intervention participants.

b Study effects based on general linear models and adjusted for age, education, baseline value of the outcome, and self-efficacy for fruit and vegetable consumption.

c Highest possible number of fruits was 14 and highest possible number of vegetables was 25.

All but 4 of the 21 intervention participants interviewed reported challenges to picking up the produce box. Work schedules (n = 8), transportation (n = 4), and forgetting (n = 4) were the most common challenges. Distance, health issues, and out-of-town travel were also challenges. Similar challenges were listed for attending evening skill-building sessions; night classes and family activities often conflicted with evening attendance. The community agency had problems contacting participants with reminders because participants “graduated” from agency classes, and contact information changed frequently.

All intervention participants reported that they would be willing to pay $10 per week for a produce box in the future. Many were willing to pay more: 15 (71%) were willing to pay $15, 8 (38%) were willing to pay $20, and 3 (14%) were willing to pay $25. Of the 9 who reported receiving WIC (Special Supplemental Nutrition Program for Women, Infants, and Children) or SNAP (Supplemental Nutrition Assistance Program) benefits, 7 said they would be willing to use these benefits for the produce.

In an overall evaluation of the Farm Fresh Healthy Living program, 20 of 21 participants stated that if the program were conducted again, they would participate. In an open-ended question asking “What did you think of the program?,” all 21 responded positively. Responses included mention of the variety provided weekly, the chance to eat foods that were too expensive to purchase at the grocery store, the chance to expose children to new foods, and the better flavor of local produce compared with grocery store produce.

Debriefing results identified problems and possible solutions (Figure). Clarifying agency expectations and siting the program in agencies that are natural hubs of family activity (eg, community centers, day- or afterschool care centers) could improve participation and maintaining participant contact. Adapting produce box contents and size to participant preferences could make the program more effective and sustainable. More sensitive and specific evaluation methods could provide better understanding of the program’s impact on the overall household food system.

**Figure:**
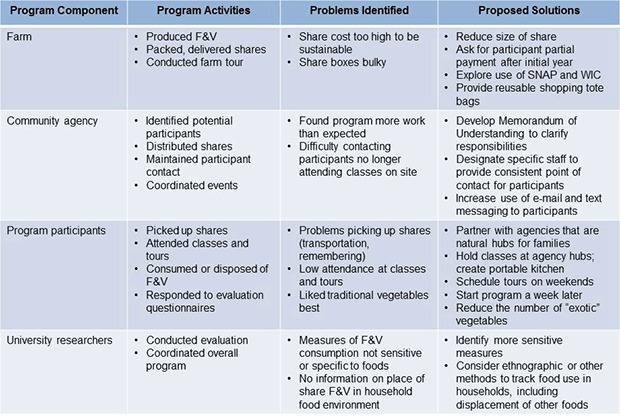
Results of process evaluation, Farm Fresh Healthy Living feasibility study, Forsyth County, North Carolina, 2012. Evaluation indicated problems identified and solutions proposed for follow-up study; some proposed solutions are indicated for more than 1 problem area. Abbreviations: F&V, fruits and vegetables; SNAP, Supplemental Nutrition Assistance Program; WIC, Special Supplemental Nutrition Program for Women, Infants, and Children.

## Discussion

This study was designed to evaluate the effects of supplying local food from a CSA program to low-income families on families’ reported household food inventory and fruit and vegetable consumption. Compared with a control group, the participants in the Farm Fresh Healthy Living program reported a greater variety of fruits and vegetables in their households at the end of the season than did the control group. There was no difference in fruit and vegetable intake, though a trend toward higher consumption in the intervention group was observed. The greater number of fruits and vegetables present in food inventories of intervention participants demonstrates that the intervention increased the diversity of foods available to families. A larger sample size or a more sensitive data collection instrument for dietary intake may have demonstrated a significant effect on fruit and vegetable consumption.

Participants picked up their food an average of 9 of 16 weeks. Although the pick-up schedule was more generous than is the case with other CSA programs and accommodations were made for participants’ schedules, these efforts may have been insufficient to adjust the program to the life situations of low-income women. Many women worked multiple jobs and lacked workplace flexibility. Some depended on multiple buses to travel to and from the pick-up point, and they struggled to transport the produce box on the bus. Such issues that prevented full participation are consistent with those observed by other researchers (17,24). Proposed program changes reflect the problems of promoting and maintaining participation, increasing sustainability, and targeting outcome evaluation. Although participants reported liking and using most foods, whether such foods supplement regularly eaten foods (as intended) or displace them should be investigated. 

Nationally, the proportion of fruits and vegetables consumed fresh has increased over the last 2 decades from 40% to 46% (25). However, substantial disparities exist in fresh produce consumption; households with incomes of $100,000 or more average $712 spent per capita in 2009, compared with $254 per capita spent by those with incomes less than $15,000 (25). These disparities in consumption, as well as the growing literature on food access disparities, underlie attempts to improve the food environment for underresourced communities. These efforts have been diverse, including supporting urban agriculture, incentivizing new grocery stores, promoting healthy corner stores, and establishing new farmers markets. Expanding CSA programs to underresourced communities fits with such efforts. The limited literature on CSA programs as an alternative food source suggests that the traditional organization of CSA programs is designed primarily to support growers (26) and that most CSA members have high incomes (27). Improving food supplies to low-income communities requires factors — low prices, distributed rather than lump-sum payments, and liberal food pick-up opportunities — that run counter to a grower-first orientation. Although the participants in this study placed high value on the food provided and stated a willingness to pay per week for the food, the amount they were willing to pay was less than half what other CSA members were charged during the same summer.

Our study has limitations. This was a small feasibility study conducted with clients of a single agency, receiving food from 1 CSA program. Therefore, the results may not be representative of other populations or other CSA programs. The outcomes were measured by self-reports rather than by more detailed dietary intake measures or by household inventory observations. The fruit and vegetable consumption measure focused on the mother, so the effect of the CSA share on others in the family is unknown. Not all possible explanatory factors (eg, distances from home to pick-up) were measured.

Despite these limitations, the study has several strengths. The design incorporated a well-matched control group drawn from the same population. The outcome measures have been widely used, particularly that for fruit and vegetable consumption, which is derived from the BRFSS evaluation (23). The statistical analysis adjusted for potential confounders, including education and self-efficacy for fruit and vegetable consumption. Process measures were used to identify problems and possible solutions that will be implemented in a follow-up intervention.

Expanding access to healthful foods is an important step in reducing health disparities. This study shows that food from a CSA program has positive effects on recipient households. Altering some of the financial and operational aspects of traditional CSA programs will be necessary to improve the impact of CSA participation.
